# Epigenetic regulation of thermomorphogenesis in *Arabidopsis thaliana*

**DOI:** 10.1007/s42994-022-00070-9

**Published:** 2022-03-14

**Authors:** Yifeng Hou, Yan Yan, Xiaofeng Cao

**Affiliations:** 1 State Key Laboratory of Plant Genomics and National Center for Plant Gene Research, Institute of Genetics and Developmental Biology, Chinese Academy of Sciences, Beijing, 100101 China; 2 University of Chinese Academy of Sciences, Beijing, 100049 China; 3 Center for Excellence in Molecular Plant Sciences, Chinese Academy of Sciences, Beijing, 100101 China

**Keywords:** Ambient temperature, Thermomorphogenesis, Histone modification, Histone variants, Non-coding RNAs

## Abstract

Temperature is a key factor in determining plant growth and development, geographical distribution, and seasonal behavior. Plants accurately sense subtle changes in ambient temperature and alter their growth and development accordingly to improve their chances of survival and successful propagation. Thermomorphogenesis encompasses a variety of morphological changes that help plants acclimate to warm environmental temperatures. Revealing the molecular mechanism of thermomorphogenesis is important for breeding thermo-tolerant crops and ensuring food security under global climate change. Plant adaptation to elevated ambient temperature is regulated by multiple signaling pathways and epigenetic mechanisms such as histone modifications, histone variants, and non-coding RNAs. In this review, we summarize recent advances in the mechanism of epigenetic regulation during thermomorphogenesis with a focus on the model plant *Arabidopsis thaliana* and briefly discuss future prospects for this field.

## Introduction

Temperature plays an important role in plant growth and development and ambient temperature affects the entire plant life cycle (Ding et al. 2020; Lippmann et al. 2019). For example, high ambient temperatures accelerate plant growth, promote flowering, reduce seed production, and reduce disease resistance (Lee et al. 2020; Li et al. 2018). Therefore, changes in ambient temperature due to global climate change have major implications for agriculture and natural ecosystems. Indeed, the latest assessment from the UN’s Intergovernmental Panel on Climate Change (IPCC) indicates that global temperatures will continue to rise in the future (Tollefson 2021). Researchers have estimated that the yields of wheat, rice, maize, and soybean will decrease by 6%, 3.2%, 7.4%, and 3.1% for each degree-Celsius increase in global average temperature (Zhao et al. 2017). Therefore, exploring the mechanisms by which temperature regulates plant growth and development will facilitate the breeding of new crop varieties that tolerate high temperature to improve food security.

Plant growth and development are highly plastic, which helps plants survive the constantly changing external environmental temperatures. At high ambient temperature (below the heat-stress range), plants undergo adaptive growth, which leads to a variety of morphological changes termed thermomorphogenesis (Casal and Balasubramanian 2019; Quint et al. 2016). Typical thermomorphogenesis phenotypes of *Arabidopsis thaliana* include hypocotyl elongation, petiole lengthening, and hyponastic growth of petiole and leaf (Casal and Balasubramanian 2019; Quint et al. 2016).

Plants have evolved intricate and accurate systems for sensing temperature and transmission of temperature signals; these systems quickly sense changes in the surrounding environment and make corresponding adjustments, thus enhancing plant tolerance to ambient temperature. Thermomorphogenesis is regulated by the integration of signals from the ambient temperature, light, hormonal, and circadian clock pathways (Casal and Balasubramanian 2019; Ding et al. 2020). In addition, epigenetic regulatory mechanisms play an important role in plant temperature responses (Casal and Balasubramanian 2019; Chang et al. 2019; He et al. 2021a; Zhao et al. 2021).

Epigenetic regulatory mechanisms are complicated and diverse, including covalent modifications of DNA and histone tails, histone variants, and non-coding RNAs (Allis and Jenuwein 2016; Boquete et al. 2021). Epigenetic mechanisms regulate many crucial cellular processes including transcription and RNA metabolism, thus affecting growth and development (Gallusci et al. 2017; Martire and Banaszynski 2020; Ueda and Seki 2020). Many epigenetic regulatory factors and chromatin modifications are involved in the plant response to stress, which are important for plants to acclimate to harmful environmental conditions (Chang et al. 2019; He et al. 2021a; Meyer 2015; Ueda and Seki 2020). In this review, we discuss recent progress in understanding the epigenetic mechanisms that regulate thermomorphogenesis in *Arabidopsis*, potential research priorities, and their implications for crop breeding.

## Histone modification

Various post-translational modifications of core histones, including methylation and acetylation, participate in the complex mechanisms regulating transcription in plants (He et al. 2021a; Ueda and Seki 2020). Histone methylation occurs primarily on the lysine (K) and arginine (R) residues of the core histone tail, including mono-, di, and tri-methylation of H3K4, K9, K27, and K36 (Grosselin et al. 2019; Ueda and Seki 2020). Histone methylation plays an important role in regulating the expression of temperature-sensitive genes, particularly methylation of H3K4, H3K36, and H3K27.

### Histone methylation is associated with transcriptional activation in thermomorphogenesis

H3K4me3 and H3K4me2 are usually associated with gene activation (Zhang et al. 2009) and H3K4 methylation is dynamically regulated. JUMONJI C (JmjC) domain-containing proteins (JMJ) are a class of important histone demethylases (Lu et al. 2008). For example, the histone H3K4m3 demethylases JMJ14, JMJ15, and JMJ18 demethylate histone H3K4me3 associated with a cluster of genes, thus mediating their downregulation at warmer temperatures (Cui et al. 2021).

Other epigenetic regulators are also involved in regulating H3K4 methylation of specific genes in response to warm temperatures. The RNA-binding protein FLOWERING CONTROL LOCUS A (FCA) regulates H3K4me2 demethylation in *FLOWERING LOCUS C* (*FLC*) to promote flowering and restricts H3K4me2 on the auxin biosynthesis-related gene *YUCCA8* (*YUC8*) promoter (Lee et al. 2014; Liu et al. 2007). PHYTOCHROME INTERACTING FACTOR 4 (PIF4), the basic helix-loop-helix transcription factor in *Arabidopsis*, plays a centre role in thermomorphogenesis (Koini et al. 2009). PIF4 promotes high temperature-induced hypocotyl elongation by binding to the promoter region of *YUC8* to activate *YUC8* expression (Sun et al. 2012). Moreover, FCA promotes the dissociation of PIF4 from the *YUC8* promoter by modifying H3K4me2 level at *YUC8* chromatin, thus limiting thermal acceleration of stem growth (Lee et al. 2014). The transcription factor SEUSS (SEU), a positive regulator of thermomorphogenesis, mediates the enrichment of H3K4me3 in the chromatin regions of *YUC8* and *INDOLE-3-ACETIC ACID INDUCIBLE 19* (*IAA19*) to promote hypocotyl elongation in warmer temperature (Huai et al. 2018). Chromatin remodeling factors such as INOSITOL REQUIRING 80 (INO80) have important roles in regulating H3K4 methylation response to temperature. The *atino80* mutants displayed strong reductions of hypocotyl and petiole elongation under high ambient temperatures (Xue et al. 2021). The INO80 chromatin remodeling complex (INO80-C) promotes H3K4me3 deposition and transcript elongation at PIF4 targets to regulate gene expression in response to warm temperature (Xue et al. 2021).

Histone H3 lysine 36 trimethylation (H3K36me3) is an important marker for transcriptional elongation and positively regulates transcription rate (Wagner and Carpenter 2012). At warm temperatures, H3K36me3 levels tend to be higher and present in broader regions (Pajoro et al. 2017). H3K36me3 mediated by SET DOMAIN GROUP8 (SDG8) and SDG26 is required for temperature-induced alternative splicing, and mutations of SDG8 and SDG26 impair warmer temperature-induced flowering in *Arabidopsis* (Pajoro et al. 2017). MORF-RELATED GENE 1 (MRG1) and MRG2 are involved in H3K36me3 binding (Xu et al. 2014) and the *mrg1-1mrg2-3* double mutants show reduced temperature-induced flowering, further demonstrating that H3K36 plays a regulatory role in plant responses to fluctuating ambient temperatures (Pajoro et al. 2017).

### Histone methylation is associated with transcriptional inhibition in thermomorphogenesis

H3K27me3 marks are located in gene bodies of genes with high and low transcription rates and negatively regulate these genes under warm temperatures (Sidaway-Lee et al. 2014). JMJ13 and JMJ30/JMJ32 negatively regulate temperature-dependent flowering (Gan et al. 2014; Zheng et al. 2019). Warm temperature induces the expression of *JMJ30* and *JMJ13*, thus facilitating their protein accumulation (Gan et al. 2014; Zheng et al. 2019). JMJ30 directly binds to the *FLC* locus, removing H3K27me3, promoting *FLC* expression, and thereby inhibiting warm temperature-induced flowering (Gan et al. 2014). JMJ13 has two homologs: EARLY FLOWERING 6 (ELF6/JMJ11) and RELATIVE OF EARLY FLOWERING 6 (REF6/JMJ12). REF6 recognizes a CTCTGYTY motif via its four Cys2His2 zinc fingers domains and is mainly involved in the demethylation of H3K27me2 and H3K27me3; moreover REF6 activity is affected by non-CG DNA methylation (Cui et al. 2016; Lu et al. 2011; Qiu et al. 2019). REF6 and HEAT SHOCK FACTOR A2 (HSFA2) form a heritable transcriptional feedback loop that regulates several processes, such as flowering and immunity, in response to heat stress (Liu et al. 2019). Recent studies have shown that hypocotyl elongation of *ref6* mutants was inhibited under warm ambient temperatures and REF6 enzyme activity is essential for plant response to this process. REF6 and PIF4 synergistically activate the expression of thermo-responsive genes at warm ambient temperature, thereby participating in the regulation of thermomorphogenesis (He et al. 2021b). In addition, the ATP-dependent chromatin remodeling factor PICKLE (PKL) down regulates H3K27me3 at *IAA19* and *IAA29*, promoting gene expression in a temperature-dependent manner (Zha et al. 2017). *pkl* mutations lead to reduced sensitivity of hypocotyl elongation to warm temperatures, suggesting that PKL plays an important role in regulating thermomorphogenesis (Zha et al. 2017). However, the relationship of REF6 and PKL is still unknown, needs to be further addressed.

### Histone deacetylation in thermomorphogenesis

Histone acetylation and deacetylation play important roles in plant growth, development, and environmental adaptation (Chen et al. 2020a; Liu et al. 2014; Ma et al. 2013). Histone acetylation and deacetylation are reversible and dynamically regulated by histone acetyltransferases (HATs) and histone deacetylases (HDACs) (Chen et al. 2020a). HDACs are ubiquitous and relatively well conserved in eukaryotes. Recent studies have gradually begun to reveal the mechanisms by which histone deacetylation regulates gene expression and responds to environmental temperature changes.

Three histone deacetylases, HDA9, HDA15 and HDA19, are involved in thermomorphogenesis of *Arabidopsis*. At high ambient temperatures, the *hda9* mutant has shorter hypocotyls compared to the wild type, suggesting that the deletion of HDA9 resulted in a weaker response to warm temperatures (Shen et al. 2019; van der Woude et al. 2019). Notably, the impaired thermomorphogenesis in *had9* mutants is independent of light-quality signaling and phytochrome B (phyB) (van der Woude et al. 2019). The expression level of several warm-temperature marker genes was down-regulated in *hda9-1* mutants compared with the wild type at 27 °C, which further confirmed that the *hda9* mutant was insensitive to warm temperature (Shen et al. 2019). However, no direct binding or significant enrichment of HDA9 was detected on several warm-temperature marker genes (*HSP20*, *HSP20-like*, *IAA19*, *IAA29*, and *bZIP63*) (Shen et al. 2019), suggesting that HDA9 is not directly associated with these genes. HDA9 does not directly bind to these warm-temperature marker genes, but it is necessary for their expression, suggesting that HDA9 may regulate the expression of warm-temperature marker genes through other mechanisms at elevated ambient temperatures.

HDACs form histone deacetylase complexes (Chen et al. 2020a). For example, the SANT (SWI3/DAD2/N-CoR/TFIII-B) domain protein POWERDRESS (PWR) interacts with HDA9 and is required for HDA9 function (Chen et al. 2016; Kim et al. 2016). The *pwr* and *had9* mutants showed highly similar phenotypes, including impaired thermomorphogenesis (Mayer et al. 2019; Tasset et al. 2018; van der Woude et al. 2019). Warm temperatures induce H3K9 deacetylation at the + 1 nucleosomes of *PIF4* and *YUC8*, but in *pwr* mutants, these nucleosomes exhibit H3K9 hyperacetylation at warm temperatures (Tasset et al. 2018). Similarly, in *had9* mutants, warm temperature induced an increase in H3K9 acetylation at the transcriptional start site and gene body of *YUC8*, demonstrating the importance of histone deacetylation in thermomorphogenesis (van der Woude et al. 2019).

Similar to the *hda9-1* mutant, the *hda19* mutants also had shorter hypocotyls than that of the wild type at high ambient temperatures and downregulated expression of several warm-temperature marker genes (*HSP20*, *HSP20-like*, *IAA19*, *IAA29* and *bZIP63*), indicating a diminished response to warm temperatures (Shen et al. 2019). However, unlike HDA9, HDA19 can bind to the exon and/or promoter regions of several warm-temperature marker genes (*HSP20*, *HSP20-like*, *IAA19*, *IAA29*, and *bZIP63*), and it appears that HDA19 binding is related to the expression of these genes. HDA19 inhibits photomorphogenesis in *Arabidopsis* (Benhamed et al. 2006; Jing et al. 2021). Compared with the wild-type, the *hda19-2* mutant showed an enhanced photomorphogenesis phenotype, with shorter hypocotyls under far-red (FR), red light (RL) and blue light (BL) conditions (Benhamed et al. 2006; Jing et al. 2021). Considering that HDA19 functions in both thermomorphogenesis and photomorphogenesis, it seems that HDA19 may be involved in the regulation of hypocotyl elongation through the integration of light and temperature signals.

Recent studies have shown that HDA15 is a direct repressor of *Arabidopsis* responses to elevated ambient temperature, and seedlings of *hda15-1* mutants show enhanced high ambient temperature responses (Shen et al. 2019). Compared with wild-type Col-0 plants, *hda15-1* seedlings showed a long hypocotyl phenotype at 20 °C and 27 °C; in particular, the hypocotyls of *hda15-1* seedlings were significantly longer than those of Col-0 at high ambient temperatures, indicating that *hda15-1* seedlings showed a hyper-response to elevated ambient temperature. Transcriptome analysis showed that *hda15-1* mutants induced the expression of some warm temperature-induced genes at 20 °C, and the expression of these genes was more upregulated at high ambient temperature, indicating that HDA15 negatively regulates the expression of warm temperature-induced genes. Further analysis revealed that HDA15 interacts with the transcription factor LONG HYPOCOTYL IN FAR RED1 (HFR1) to synergistically inhibit temperature responses (Shen et al. 2019).

Three HDACs (HDA9, HDA15, and HDA19) are reported to be involved in thermomorphogenesis. Interestingly, different HDACs have different or even opposite functions in thermomorphogenesis (Shen et al. 2019). Analysis of the differentially expressed target genes revealed that different HDACs regulate hypocotyl elongation by distinct mechanisms, indicating the complexity and diversity of HDAC functions in thermomorphogenesis. The functions of HDACs depend to some extent on their interacting proteins, which may be one of the reasons for the differential response of HDACs to elevated ambient temperature. Screening and identifying more HDAC-interacting proteins and studying the role of the complexes formed by HDAC and HDAC-interacting proteins in the warm temperature response will help to reveal the specific mechanism by which HDACs participate in thermomorphogenesis. In addition, current research has mostly focused on revealing the roles of HDACs in warm temperature responses and analyzing the target genes regulated by HDACs, while the effects of HDACs on histone acetylation and chromatin state are not clear. Analyzing the effect of HDAC deletion on the acetylation level of specific histone sites and comparing the changes of histone acetylation in response to warm temperature will help to reveal the mechanism by which HDACs regulate the expression of temperature-responsive genes.

## H2A.Z nucleosomal dynamics

H2A.Z is a conserved variant of histone H2A present in most eukaryotic organisms. In most cases, H2A.Z is enriched on the first nucleosome downstream of the transcription start site (TSS + 1 nucleosome) and in the gene body, causing nucleosomes to wrap DNA more tightly, which influences the ability of RNA polymerase (Pol) II to transcribe genes in plants (Cortijo et al. 2017; Kumar and Wigge 2010; Xue et al. 2021). H2A.Z is incorporated into chromatin to replace H2A by the SWI2/SNF2‐RELATED 1 complex (SWR1c), composed of ACTIN‐RELATED PROTEIN 6 (ARP6), ARP4, SWR1 COMPLEX 4 (SWC4), SERRATED LEAVES AND EARLY FLOWERING (SEF) and PHOTOPERIOD‐INDEPENDENT EARLY FLOWERING 1 (PIE1) in *Arabidopsis* (Aslam et al. 2019; Choi et al. 2007; Deal et al. 2005; Gómez-Zambrano et al. 2018; Kobor et al. 2004; Krogan et al. 2003; March-Díaz et al. 2007; Mizuguchi et al. 2004; Noh and Amasino 2003).

H2A.Z loss-of-function mutants (*hta9hta11)* and *arp6* both have elevated *HEAT SHOCK PROTEIN 70* (*HSP70*) expression along with early flowering, long hypocotyls, petiole elongation, and other warm temperature-related phenotypes (Kumar and Wigge 2010). Furthermore, temperature affects the occupancy of H2A.Z, with high occupancy of H2A.Z at lower temperatures and eviction of H2A.Z at higher temperatures (Kumar and Wigge 2010). High ambient temperature induce the eviction of H2A.Z-containing nucleosomes, which contributes to the formation of an open DNA structure, thus facilitating the binding of transcription factors to activate or inhibit gene expression at high temperature (Cortijo et al. 2017; Kumar and Wigge 2010; Xue et al. 2021). For example, temperature-induced H2A.Z nucleosome dynamics gate the accessibility of PIF4 to *FLOWERING LOCUS T* (*FT*) for transcriptional activation and control plants timing of flowering in response to temperature (Kumar et al. 2012).

However, the eviction of H2A.Z-containing nucleosomes is not directly controlled by temperature, as loss of H2A.Z in chromatin does not respond to temperature in vitro (Cortijo et al. 2017). Several factors regulate the substitution of H2A.Z in nucleosomes associated with temperature-responsive genes. One of these factors is the transcription factor HEAT SHOCK FACTOR A1 (HSFA1) (Cortijo et al. 2017). HSFA1 is part of a clade of *Arabidopsis* HEAT SHOCK FACTORs (HSFs), which are the pivotal transcriptional activators of the heat shock response in eukaryotes. As the temperature rises, HSFA1 binds to temperature-responsive genes and initiates transcription by dynamically facilitating H2A.Z loss, thus antagonizing the function of H2A.Z in response to warm temperature (Cortijo et al. 2017). Warm temperature selectively enhances the translation of another HSF protein, HSFA2, by affecting the formation of an RNA hairpin within the 5′-untranslated region of the *HSFA2* mRNA (Chung et al. 2020). However, how HSFs regulate the eviction of H2A.Z-containing nucleosomes at temperature-responsive genes remains to be studied.

In addition, other epigenetic factors regulate the response of H2A.Z to temperature. HDA9 mediates histone deacetylation at *YUCCA8* and regulates temperature-induced H2A.Z eviction from nucleosomes at PIF4 targets, but does not affect the expression of *PIF4* and *HSP70*, suggesting that nucleosome dynamics and histone deacetylation are coupled during warm-temperature responses (van der Woude et al. 2019). Another important regulatory factor is the chromatin-remodeling factor INO80. The INO80 chromatin remodeling complex directly interacts with PIF4 to promote temperature-induced H2A.Z eviction at PIF4 targets, activating the expression of thermo-responsive and auxin-related genes (Xue et al. 2021). INO80 also interacts with H2A.Z, positively regulating H2A.Z-containing nucleosomes at the key flowering repressor gene *FLC* and regulating H2A.Z-mediated gene expression in response to light, thereby controlling plant growth and development (Yang et al. 2020; Zhang et al. 2015a, b). A recent study showed that PIFs (PIF4/PIF5/PIF7) undergo rapid regulation to reshape the H2A.Z and H3K9ac epigenetic landscape by interacting with EIN6 ENHANCER (EEN), a subunit of the INO80 chromatin-remodeling complex, in response to low R:FR conditions. The PIFs-INO80 module may be a hub allowing plants to regulate their growth by adapting to environmental changes at the epigenetic level (Willige et al. 2021). Light and temperature usually change in concert under natural conditions and the light and temperature signaling pathways usually share the same downstream regulatory elements.

Phytochrome B (phyB), the dominant red-light photoreceptor, exists in two photo-interconvertible forms, Pr (R light-absorbing, biologically inactive) and Pfr (FR light-absorbing, biologically active) (Li et al. 2011; Reed et al. 1993; Somers et al. 1991). PhyB also functions as a thermosensor, undergoing temperature-dependent reversion from the active Pfr form to the inactive Pr form (Jung et al. 2016; Legris et al. 2016). The Pfr form of phyB interacts with SWC6 and ARP6, positively regulating H2A.Z deposition at *YUC9* and thus mediating the inhibition of hypocotyl growth under red light (Mao et al. 2021; Wei et al. 2021). Therefore, phyB may regulate H2A.Z eviction in response to warm temperature. In addition to phyB, the blue light (BL) photoreceptor cryptochrome 1 (CRY1) has been reported to act in a similar mechanism for H2A.Z regulation under blue light. CRY1 regulates H2A.Z deposition at ELONGATED HYPOCOTYL 5 (HY5) target genes by interacting with SWC6 and ARP6, thus positively regulating photomorphogenesis (Mao et al. 2021). CRY1 and CRY2 play important roles in temperature signal transduction. CRY1 directly interacts with PIF4 to inhibit its transcriptional activity in a blue light-dependent manner, thus regulating hypocotyl elongation (Ma et al. 2016) and CRY2 accumulates at higher temperature and is degraded at lower temperature (Li et al. 2021; Ma et al. 2021).

Since both phyB and CRY1 regulate the accumulation of H2A.Z at specific sites through similar mechanisms, and they play important roles in warm temperature signal transduction, it will be interesting to investigate the mechanism by which these photoreceptors regulate H2A.Z occupancy at ambient temperatures. In addition, the NUCLEAR FACTOR‐Y subunit C (NF-YC), also known as HISTONE‐ASSOCIATED PROTEIN5 (HAP5) or CCAAT BINDING FACTOR C (CBF‐C) has been reported to regulate light‐induced H2A.Z deposition associated with hypocotyl elongation‐related genes, repressing related gene expression in a light‐dependent manner (Zhang et al. 2021). Due to the close relationship between light and temperature signaling, NF-YC may also be a potential regulatory factor for the eviction and deposition of H2A.Z at warmer temperatures.

**Fig. 1:**
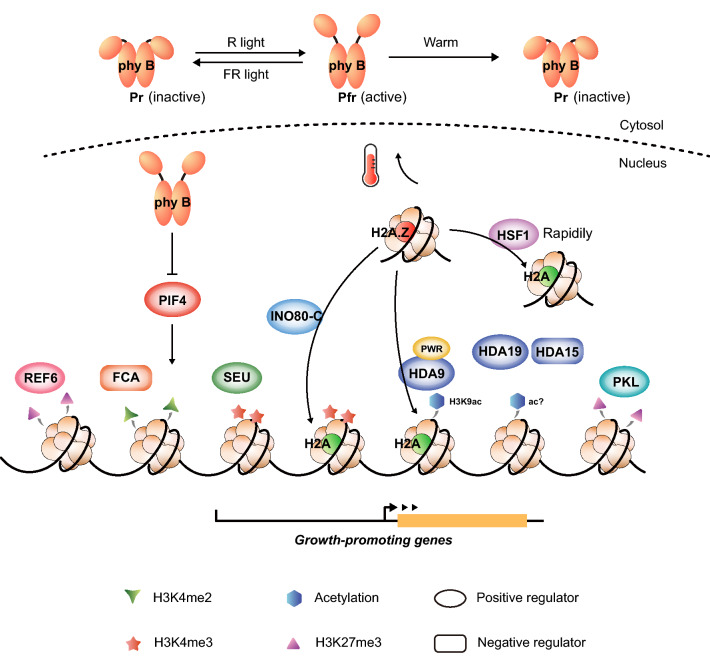
Roles of histone modifications and histone variants in regulating thermomorphogenesis in *Arabidopsis thaliana*. (i) Phytochrome B is not only the main red light sensor, but also a thermosensor. Under red light, phyB is transformed into a Pfr homodimer, enters the nucleus, and blocks PIF4 activity. Warm temperature promotes the reversion of phyB back to its inactive Pr form, releasing PIF4 to promote downstream temperature response gene expression. (ii) RELATIVE OF EARLY FLOWERING 6/JUMONJI DOMAIN-CONTAINING PROTEIN 12 (REF6/JMJ12) mediates H3K27me3 demethylation at several PIF4 target genes to positively regulate thermomorphogenesis. (iii) FLOWERING CONTROL LOCUS A (FCA)-mediated inhibition of H3K4me2 deposition results in inhibited SEUSS (SEU)-mediated enrichment of H3K4me3, which facilitates hypocotyl elongation at warmer ambient temperatures. (iv) At warmer ambient temperatures, PICKLE (PKL) mediates the downregulation of H3K27me3 levels, which promotes hypocotyl elongation. The INO80 chromatin remodeling complex (INO80-C) promotes H3K4me3 deposition and stimulates hypocotyl elongation in response to warm temperature. (v) The histone deacetylases HDA9 and HDA19 stimulate thermomorphogenesis but HDA15 directly represses plant responses to high ambient temperature. PWR, a SANT-domain containing protein known to interact with HDA9, can also stimulate thermomorphogenesis. (vi) INO80-C, HDA9 and HEAT SHOCK FACTOR A1 (HSFA1) accelerate warm temperature-induced eviction of the histone variant H2A.Z and stimulate hypocotyl elongation. (vii) FCA, SEU and INO80-C interact with PIF4, and regulate histone modifications and histone variants at PIF4 targets. The stars and triangles represent different histone methylations; the hexagons represent histone acetylation. Positive regulators of thermomorphogenesis are represented as ellipses and negative regulators as rectangles

The important role of histone modification and histone variants in plant thermomorphogenesis is gradually emerging (See Fig. 1 and Table 1). Although many specific mechanisms need to be further elucidated, this emerging picture undoubtedly provides a new perspective on the mechanisms by which plants respond to environmental temperature. In addition to revealing the mechanism by which single epigenetic markers participate in thermomorphogenesis, we should also consider crosstalk between epigenetic markers in thermomorphogenesis. At warm temperatures, HDA9 mediates histone deacetylation of YUC8 and induces eviction of H2A.Z from nucleosomes at the YUC8 locus, suggesting that histone variants and histone deacetylation act in the temperature response (van der Woude et al. 2019). A recent report finds that INO80 is involved in promoting thermomorphogenesis by integrating H2A.Z eviction and H3K4me3 deposition (Xue et al. 2021). Recent studies have also shown that PIF transcription factors affect the H2A.Z and H3K9ac landscape in a light-quality-dependent manner (Willige et al. 2021). In addition, phyB has been reported to directly interact with VERNALIZATION INSENSITIVE 3-LIKE1/VERNALIZATION 5 (VIL1/VRN5), the core component of Polycomb Repressive Complex 2, to promote the enrichment of H3K27me3 in a light-dependent manner (Kim et al. 2021); furthermore, the direct interaction of phyB‐SWC6/ARP6 promotes the deposition of H2AZ at *YUC9*, thereby inhibiting hypocotyl elongation in red light (Wei et al. 2021). Considering that light signaling regulates thermomorphogenesis, the changes in H2A.Z and histone modifications may occur as part of crosstalk in response to ambient temperature. Such crosstalk between epigenetic markers may act as a fine-tuning mechanism to regulate plant responses to subtle changes in ambient temperature. Decoding how chromatin landscapes change in response to temperature changes, and thus rapidly fine-tune gene expression, will play a key role in uncovering the mechanisms by which plant morphogenesis responds to environmental challenges.

**Table 1:** Summary of epigenetic regulation of thermomorphogenesis in *Arabidopsis thaliana*

Mutants	Epigenetic regulatory mechanisms	Temperature ranges (low/high)	The *Arabidopsis* accessions used	The duration of treatment	The resulting phenotypes	References
*icdh/jmj14*	JMJ14 is an H3K4me3 demethylase	20 °C/27 °C	Col	For hypocotyl length measurement, seedlings were grown at 20 °C for 4 days under SD conditions and further grown at either 20 °C or 27 °C for 3 days. For leaf angle measurement, 7-day-old seedlings grown at 20 °C then exposed to 27 °C for 4 days	Shorter hypocotyl and reduced leaf angles at 27 °C	Cui et al. (2021)*, Lu et al. (2010), Yang et al. (2018), Zhang et al. (2015b)
*fca*	FCA as an RNA binding protein mediates the demethylation of H3K4me2	23 °C/28 °C	Col	Seedlings were grown at 23 °C for 4 days under continuous light conditions and further grown at either 23 °C or 28 °C for 3 days	Elongated hypocotyls, increased leaf hyponasty and elongated leaf stem (petiole) at 28 °C	Lee et al. (2014)*, Macknight et al. (1997)
*seu*	SEU as a transcription regulator mediates the enrichment of H3K4me3	22 °C/28 °C	Col	Seedlings were grown under continuous white light at 22 °C or 28 °C for 5 days	Shorter hypocotyl at 28 °C	Franks et al. (2002), Huai et al. (2018)*
*ino80*	INO80 is a chromatin remodeler that regulate H2A.Z eviction	21 °C/27 °C	Col	Seedlings were grown at 21 °C under SD conditions for 4 days and further grown at either 21 °C or 27 °C for 4 days	Shorter hypocotyl at 27 °C	Xue et al. (2021)*, Zhang et al. (2015a)
*sdg8*	SDG8 is an H3K36me3 methyltransferase	16 °C/25 °C	Col	Seedlings were grown at 16 °C constantly or moved to 25 °C after five weeks	Compromised temperature induced-flowering	Pajoro et al. (2017)*, Xu et al. (2008), Zhao et al. (2005)
*sdg26*	SDG26 is an H3K36me3 methyltransferase	16 °C/25 °C	Col	Seedlings were grown at 16 °C for five weeks and then maintained at 16 °C or transferred to 25 °C	Compromised temperature induced-flowering	Pajoro et al. (2017)*, Xu et al. (2008)
*jmj13*	JMJ13 is an H3K27me3 demethylase	22 °C/28 °C	Col	Seedlings were grown under the standard growth conditions (LD 22 or LD 28 °C)	Flowers early in LD conditions regardless of temperature	Lu et al. (2008), Zheng et al. (2019)*
*jmj30* *jmj32*	JMJ30 mediate H3K27me2/3 demethylation	22 °C/29 °C	Col	Seedlings were grown under the standard growth conditions (LD 22 or LD 29 °C)	Accelerated flowering at 29 °C	Gan et al. (2014)*, Lu et al. (2008)
*ref6*	REF6 is an H3K27me2/3 demethylase	22 °C/28 °C	Col	Seedlings were grown at 22 °C under LD conditions for 3 days and further grown at either 22 °C or 28 °C for 3 days	Attenuated hypocotyl elongation at 28 °C	Cui et al. (2016), He et al. (2021b)*, Li et al. (2016), Lu et al. (2011), Tian et al. (2020)
*pkl*	PKL is an ATP‐dependent chromatin remodeling factor that mediates the removal of H3K27me3	22 °C/28 °C	Col	Seedlings were grown at 22 °C under continuous white light for 96 h and further grown at either 22 °C or 28 °C for 3 days	Reduced hypocotyl elongation at 28 °C	Ho et al. (2013), Zha et al. (2017)*
*hda9*	HDA9 is a histone deacetylase	20 °C/27 °C	Col	Upon germination at 20 °C, seedlings were grown at either 20 °C or 27 °C under SD conditions for 7 days. (Shen et al. 2019) Seedlings were grown at either 22 °C or 27 °C under SD conditions for 8 days (van der Woude et al. 2019)	Shorter hypocotyl at 27 °C	Pandey et al. (2002), Shen et al. (2019)*, van der Woude et al. (2019)*
*hda19*	HDA19 is a histone deacetylase	20 °C/27 °C	WS	Upon germination at 20 °C, seedlings were grown at either 20 °C or 27 °C under SD conditions for 7 days	Shorter hypocotyl at 27 °C	Jing et al. (2021), Pandey et al. (2002), Shen et al. (2019)*
*hda15*	HDA15 is a histone deacetylase	20 °C/27 °C	Col	Upon germination at 20 °C, seedlings were grown at either 20 °C or 27 °C under SD conditions for 7 days	Longer hypocotyl at 27 °C	Chen et al. (2020b), Pandey et al. (2002), Shen et al. (2019)*
*arp6*	ARP6 is a chromatin remodeling factor that mediates H2A.Z occupancy	17 °C, 22 °C and 27 °C	Col	Short days	Thermal induction of flowering at 27 °C; elongated hypocotyl and enhanced petiole growth at 17 °C, 22 °C and 27 °C	Kumar and Wigge (2010)*, Martin-Trillo et al. (2006), Wei et al. (2021)
*miR172a*	*miR172a* is a small RNAs	16 °C/23 °C	Col	Plants were grown under LD-conditions	*miR172aOX* exhibited temperature-independent early flowering	Chen (2004), Lee et al. (2010)*
*flinc*	*FLINC* is a long non-coding RNA	16 °C/25 °C	Col	Plants were grown at 16 °C in growth cabinets in LD conditions for 3 weeks and further grown at either 16 °C or 25 °C	Less sensitive to temperature-mediated flowering	Severing et al. (2018)*

References related to temperature treatment and corresponding phenotypes is marked with an asterisk [*]

## Non-coding RNAs and RNA metabolism

Small RNAs (single-stranded 21–24 nucleotide RNAs) regulate the post-transcriptional expression of genes and include microRNAs (miRNAs) and small interfering RNAs (siRNAs). Small RNAs play key roles in regulating plant growth and development, signal transduction, stress responses, and genome stability (D'Ario et al. 2017; Deng et al. 2016; Si et al. 2020). In addition, the role of small RNAs in ambient temperature adaptation of plants is emerging (Gyula et al. 2018; Kim et al. 2012; Lee et al. 2010; Serivichyaswat et al. 2017). Temperature regulates the abundance of certain small RNAs and such ambient temperature-responsive miRNAs are involved in the regulation of flowering time (Gyula et al. 2018; Lee et al. 2010; Serivichyaswat et al. 2017). Although warm temperatures promoted the accumulation of miR172a, plants that overexpressed miR172a exhibited a temperature-independent early flowering phenotype, suggesting that changes in miR172a accumulation affected plant temperature perception (Lee et al. 2010). The accumulation of miR169 is down-regulated at warm temperatures, leading to the activation of NF‐YA‐dependent genes that promote flowering (Gyula et al. 2018).

Long non-coding RNAs (lncRNAs, > 200 nucleotide RNAs that are not translated into functional proteins) occur widely in eukaryotes (Statello et al. 2021; Wierzbicki et al. 2021) and regulate gene expression at the transcriptional and post-transcriptional levels (Statello et al. 2021; Wierzbicki et al. 2021). Ongoing work has identified lncRNAs whose expression responds to environmental temperature changes, suggesting that lncRNAs have potential regulatory roles in temperature-dependent plant development (Severing et al. 2018). For example, expression of the lncRNA *FLOWERING LONG INTERGENIC NON-CODING RNA* (*FLINC*) was down-regulated at warm temperatures and the *flinc* mutants flowered earlier than wild type, confirming the regulatory role of *FLINC* in thermomorphogenesis (Severing et al. 2018). However, the molecular mechanisms of the known ambient temperature-regulated lncRNAs is not clear, and their biological functions in plant thermomorphogenesis remain to be revealed.

The metabolism and processing of mRNAs is particularly important in environmental temperature responses. For example, alternative splicing modulates plant responses to high temperature (Ling et al. 2021). RNA transcription and processing are tightly coupled, providing an opportunity for epigenetic factors to regulate RNA processing (Soles and Shi 2021; Tellier et al. 2020). For example, H3K36me3 plays an important role in regulating temperature-induced alternative splicing and temperature-dependent flowering time (Pajoro et al. 2017). Indeed, the important regulatory role of epigenetic modification in RNA metabolism such as alternative polyadenylation and RNA modification is attracting increasing attention (Kan et al. 2021; Soles and Shi 2021). Moreover, temperature can affect translation: at higher temperatures, the RNA hairpin in the 5′-untranslated region of *PIF7* adopts a loose conformation, leading to enhanced translation efficiency (Chung et al. 2020). Exploring the effects of epigenetic regulatory mechanisms on RNA metabolism of key genes involved in thermomorphogenesis will deepen our understanding of plant responses to warm temperature.

## Concluding remarks and future prospects

Plant cell types have distinct functions and the synergies among different cell types determine the plasticity of plant development. Revealing the functions of cell types, even at the single-cell level, is crucial to understanding the environmental adaptation of plants (Rich-Griffin et al. 2020). High-throughput single-cell sequencing techniques developed in recent years have provided unprecedented insights into critical cellular processes. Cell type-specific transcriptome analysis has begun to reveal the basic cellular activities involved in plant development and stress adaptation (Rich-Griffin et al. 2020). It is also beginning to be possible to quantitatively measure the dynamics of transcription activity in single cells in living plants under changing environmental conditions (Alamos et al. 2021; Hani et al. 2021). However, the study of cell type-specific or single-cell epigenomics in plant species remains limited.

The role of epigenetic factors in defining individual cell types and the extent to which they vary during plant development or under environmental stress remains unknown. Epigenome studies based on single-cell behavior have tremendous potential for exploring the regulatory mechanisms of plant perception and responses to changes in environmental temperature. In addition, the explosion of single-cell genomics in recent years has created an opportunity to jointly reveal the variation and differential use of genomic information in different cell types of plant species during development, differentiation, and response to environmental stresses at multiple levels (Perkel 2021; Thibivilliers and Libault 2021). The combination of cellular epigenomics and other omics at the single-cell level will help us describe the association between cell behavior and plant thermomorphogenesis phenotypes under continuously changing ambient temperatures. Obtaining multiomic molecular information at the single-cell level will be critical to reveal the functions of individual genes and the molecular mechanisms that regulate their expression, and to develop new strategies to optimize temperature-responsive agronomic traits, ultimately achieving sustainable improvements in agricultural productivity.

Thermomorphogenesis of plants is closely related to their adaptation to global climate change, as global warming has a profound impact on plant morphology, development, and geographical distribution (Lee et al. 2020; Lippmann et al. 2019). In-depth analysis of the molecular mechanisms of thermomorphogenesis is helpful to explore ways to improve the adaptability of plants to climate change and produce climate-smart crops (Lippmann et al. 2019; Park et al. 2021). Epigenetic regulatory mechanisms are critical for plant development and phenotypic plasticity, including adaptive responses to ambient temperature. Epigenetic stability and diversity and their genetic characteristics are a new source of phenotypic variation to improve plant adaptation to changing global climate and to drive breeding to ensure crop yield and quality. Future studies on the variation characteristics of crop epigenetic markers and their association with gene expression and phenotype will expand the space for plant breeding. Considering epigenetic modification in response to environmental temperature changes will facilitate the exploration of breeding strategies needed to address food security challenges in the context of global warming.
